# Predictors of cancer screening behavior of the working population in China based on the information-motivation-behavioral skills model

**DOI:** 10.3389/fpubh.2023.1112172

**Published:** 2023-07-27

**Authors:** Zezhou Wang, Changming Zhou, Li Zhang, Jie Shen, Miao Mo, Yulian He, Ying Zheng

**Affiliations:** ^1^Department of Cancer Prevention, Fudan University Shanghai Cancer Center, Shanghai, China; ^2^Department of Oncology, Shanghai Medical College, Fudan University, Shanghai, China; ^3^Pudong New Area Center for Disease Control and Prevention, Shanghai, China; ^4^Department of Epidemiology, School of Public Health, Fudan University, Shanghai, China; ^5^Shanghai Engineering Research Center of Artificial Intelligence Technology for Tumor Diseases, Shanghai, China

**Keywords:** information-motivation-behavioral skills model (IMB), cancer screening, behavior change, working population, behavioral skills, China

## Abstract

**Background:**

The cancer screening rate in the working population is very low in China. Information-motivation-behavioral skills (IMB) model has been applied to elucidate screening behavior for various chronic diseases but has not been investigated in analyzing cancer screening behavior. This study aimed to examine factors influencing cancer screening behavior and their linkages based on the IMB model.

**Methods:**

A cross-sectional study was conducted in Shanghai, China from August to October 2021. Data were obtained through an anonymous questionnaire. Predictive relationships between variables in the IMB model and cancer screening behavior were evaluated. Structural equation modeling (SEM) was constructed to demonstrate the utility of the IMB model.

**Results:**

Among the 556 participants included in the analysis, 34.4% of participants had ever done a cancer screening. The construct validation analysis supported that the measure items included were acceptable. SEM found that knowledge of cancer warning signs and symptoms (β = 0.563, *p* < 0.001) and cancer screening behavioral skills (β = 0.264, *p* = 0.003) were related to participation in cancer screening, whereas cancer screening motivation was not directly influenced the participation in cancer screening (β = − 0.075, *p* = 0.372).

**Conclusion:**

The cancer screening rate was found to be lower than expected in the working population. The IMB model could be used to make decisions in implementing behavioral interventions to participate in cancer screening among the Chinese working population. Enhancing the knowledge of cancer warning signs and symptoms and strengthening behavioral skills should be focused on to improve participation in cancer screening.

## Introduction

Cancer is a major public health threat and a leading cause of death globally, with the occurrence of nearly 10 million deaths in 2020 (1). Despite the advancement in treatment options, the overall prognosis for advanced cancers remains poor. The physical, emotional, social, and spiritual effects of cancers on patients and families are also immeasurable (2). Screening for cancers aids in early detection, diagnosis, and treatment, with a better prognosis potential in early-stage cancers (3). Therefore, cancer screening is crucial for cancer prevention and management.

Based on previous reports, the estimated fraction of total cancer attributable to occupational hazards or factors generally ranged between 2 and 20% worldwide (4–6). In China, the working population constitutes a significant proportion of the population. According to Ministry of Human Resources and Social Security of the People’s Republic of China, by the end of 2021, the total labor force was approximately 746 million in China, including 468 million in urban areas. The working population is vital to China’s economic and social development, as they significantly contribute to the country’s gross domestic product and play a crucial role in promoting innovation and entrepreneurship. In addition to high-risk occupations where people can be chronically exposed to some carcinogens at work, more and more working personnel now face bad living habits such as long-term staying up late and irregularly unhealthy diet due to huge work pressure. These bad living habits may weaken their immunity, cause hormonal imbalance, disrupt the body’s biological clock, repress tumor suppressor genes, and stimulate tumor growth (7). Despite this, cancer screening rates in this working population are currently low (8) with few screening reports.

The concerning screening rates are mainly due to factors such as lack of knowledge, negative attitudes and beliefs, and poor access to healthcare services (9). Since 2005, the Chinese government has supported four organized cancer screening programs, namely the Cancer Screening Program in Rural Areas, Cancer Screening Program in Huaihe River Areas, Cervical Cancer and Breast Cancer Screening Program for Women in Rural Areas, and Cancer Screening Program in Urban Areas. By the end of 2016, approximately 2 million high-risk individuals had been screened, making an important contribution to improving early diagnosis rates. However, data from Cancer Screening Program in Urban Areas showed that the screening rates of high-risk groups for lung, liver, breast, stomach, esophageal, and colorectal cancers were only 35.3, 37.5, 40.3, 19.6, 20.2, and 27.9%, respectively (10). Moreover, there are few screening programs that focus specifically on working population.

To increase the proportion of cancer screening in the working population, health-promotion interventions based on social and behavioral science theories are more effective than those without a theoretical framework (11). The social and behavioral science postulates not only consistently explain how a phenomenon develops effective interventions by identifying predictors but also predict what will happen in the future to take preventive measures. Among the hypotheses that have been used in cancer screening behavior, the health belief model (HBM) was the most popular (12), followed by the theory of reasoned action (TRA) (13) and its extension, namely, the theory of planned behavior (TPB) (14, 15).

The information-motivation-behavioral skills (IMB) model was first proposed by Fisher in 1992 (16), which was drawn from the understanding of “motivation” from TRA and the concept of “self-efficacy” from the social cognitive theory. The potential factors were categorized into three components, information, motivation, and behavioral skills. Motivation referred to the attitude and support for acting or behaving. Based on the model assumption, participation in cancer screening was determined by the extent to which a person was informed about health information on cancer prevention, motivated to participate in the screening, and had the necessary behavior skills to participate in cancer screening. Possession of adequate health information coupled with a strong motivation to act on the information either directly promoted the occurrence of targeted cancer screening or indirectly influenced screening behaviors by promoting the desired behavioral skills. The information and motivation covaried (17).

The IMB model was initially developed to explore change in high-risk HIV transmission behavior (18) or preventive behavior (17), and subsequently successfully applied to many other illnesses and specific populations, like type II diabetes self-care behavior among Puerto Ricans (19), osteoporosis self-management behavior among Chinese adults (20), cancer screening intention among Koreans (21), and intention for HPV vaccination among U.S. college students (22). However, the model has not yet been used to investigate cancer screening behavior among the Chinese working population. To sum up, there is an urgent need for research to identify the predictors of cancer screening behavior among the working population and develop effective interventions and policies to increase the screening rate. This study aimed to fill this gap and examine factors influencing cancer screening behavior and their connections using the IMB model.

## Methods

### Research setting

Shanghai is a major city located in the eastern part of China. According to Shanghai Municipal Human Resources and Social Security Bureau statistics, there are 10.85 million working population in Shanghai in 2021. It is a global financial center and a hub for trade and commerce, with a rapidly growing economy and an increasing standard of living. In terms of healthcare, Shanghai has a well-developed healthcare system with a large number of hospitals, clinics, and medical professionals. The city also has a strong focus on disease prevention and health promotion, with various initiatives and policies aimed at improving the health of the population.

### Participants

This cross-sectional survey was conducted in Shanghai, China from August to October 2021, in collaboration with Xuhui Association for Science & Technology and Pudong Center for Disease Control and Prevention. A total of 556 participants were recruited during routine health education activities held through enterprises and social media. Individuals with a history of cancer or who refused to participate in the study were excluded. The sample size was calculated using the formula of N = Z_α_^2^P(1 − P)/d^2^ × deff. For an estimated proportion of the working population who undergo cancer screening of 40% (9), precision error (d) of 0.05, a confidence level of 95%, and design effect (deff) due to non-probability sampling of 1.5 (23), the minimum sample size required for this study was calculated to be 554. The working population who did not meet the exclusion criteria filled out the electronic questionnaire before the onset of health education activities to reduce bias. Informed consent was obtained before the participant started the survey.

### Measures

#### Socio-demographic characteristics

The socio-demographic characteristics collected were sex, age, marital status, and monthly income.

#### Information

Information was measured by the knowledge of cancer warning signs and symptoms (Supplementary Table S1). The knowledge of cancer warning signs and symptoms was assessed using an 11-item scale based on the core information and knowledge points of cancer prevention and control issued by the National Health Commission (e.g., “Do you think an unexplained lump or swelling could be a sign of cancer?”) with a “yes” and “no” response format. The knowledge of cancer warning signs and symptoms score was obtained by summing correct responses. Total scores ranged from 0 to 11, and a higher score indicated greater knowledge of cancer warning signs and symptoms information (Cronbach’s α = 0.95).

#### Motivation

Motivation to participate in cancer screening was assessed from the responses from five questions (Supplementary Table S2). Participants were asked to report their agreement with items (e.g., “I think physical screening is important for cancer prevention”) on a five-point Likert scale ranging from 1 (“Strongly Disagree”) to 5 (“Strongly Agree”). For unfavorable statements, the scoring was reversed. A composite score was obtained by summing the responses from the five items, with higher scores indicating more positive motivation regarding cancer screening (Cronbach’s α = 0.76).

#### Behavioral skills

Behavioral skills regarding cancer screening were assessed from five items (Supplementary Table S3). Participants reported their agreement with items [e.g., “I have access to cancer prevention and early screening information (e.g., professional media, lectures, etc.).”] on a five-point Likert scale ranging from 1 (“Strongly Disagree”) to 5 (“Strongly Agree”). A composite score was calculated by summing the responses of the five items, with higher scores indicating better positive behavioral skills regarding cancer screening participation (Cronbach’s α = 0.92).

#### Cancer screening

Participants were asked whether they ever underwent cancer screening in their lifetimes. Participants who answered “yes” were considered that they ever had a cancer screening. Cancer screening behavior in this study was defined as a formal cancer preventive screening or checkup in professional medical institutions. The screening techniques included low-dose computed tomography, ultrasound examination, gastroscopy, colonoscopy, and using tumor markers, etc.

### Statistical analyses

Descriptive statistical analysis was conducted to elucidate socio-demographic characteristics, cancer screening information, motivation and behavioral skills, and behavior. The chi-squared analysis and Mann–Whitney U test were used to examine the association between socio-demographic characteristics, cancer screening information, motivation, behavioral skills, and behavior. Spearman’s correlation analysis was performed to examine correlations among IMB model constructs. The reliability of the IMB model components was assessed by internal consistency using Cronbach’s alpha coefficient, with a coefficient of greater than 0.7 denoting good internal consistency. Confirmatory factor analysis (CFA) examined the factor structure of the scale. Once an acceptable measurement model was established, a structural equation model was built based on the IMB model. The parameters were estimated using the robust weighted least squares (WLS) approach as binary dependent variable. The direct and indirect effects were examined by a bias-corrected bootstrap procedure based on 1,000 bootstrap samples (24). Model fit was evaluated using the chi-square to degrees of freedom ratio (χ2/df), the comparative fit index (CFI), and the root mean square error of approximation (RMSEA). The ratio, χ2/df of ≤3, CFI of >0.90, and RMSEA of <0.08 indicated an acceptable model fit (25). The data analyses were performed using SPSS Statistics (version 26.0 for Windows, IBM Corp., Armonk, NY, United States) and Mplus version 8.3. All hypothesis tests were two-tailed with α = 0.05.

## Results

### Socio-demographic characteristics and cancer screening

Table 1 presents the socio-demographic characteristics of the participants. Of the 556 participants from the working population, 250 (45.0%) were males and 306 (55.0%) were females. Participants aged 31–40 years accounted for 43%. More than half of the participants were married. Participants with annual income ranging from RMB 100,000 to 200,000 were the most, accounting for 32%. Only 34.4% of all participants had ever undergone a cancer screening. Female, older, and married subjects engaged in more cancer screening participation (*p* < 0.01).

**Table 1:** The socio-demographic characteristics and their associations with cancer screening behavior (*n* = 556).

Characteristics	Number of participants	Ever did cancer screening			
		Yes	No	χ^2^	*p* value
	*N* (column %)	*N* (row %)	*N* (row %)		
Total	556 (100)	191 (34.4)	365 (65.6)		
Sex				7.14	0.008
Male	250 (45.0)	71 (28.4)	179 (71.6)		
Female	306 (55.0)	120 (39.2)	186 (60.8)		
Age (years)				18.94	<0.001
≤30	233 (41.9)	56 (24.0)	177 (76.0)		
31–40	239 (43.0)	100 (41.8)	139 (58.2)		
≥41	84 (15.1)	35 (41.7)	49 (58.3)		
Marital status				16.71	<0.001
Unmarried	236 (42.4)	59 (25.0)	177 (75.0)		
Married	313 (56.3)	128 (40.9)	185 (59.1)		
Others	7 (1.3)	4 (57.1)	3 (42.9)		
Annual income (RMB)				3.84	0.573
<100 thousand	146 (26.3)	47 (32.2)	99 (67.8)		
100–200 thousand	178 (32.0)	64 (36.0)	114 (64.0)		
200–300 thousand	116 (20.9)	39 (33.6)	77 (66.4)		
300–400 thousand	37 (6.7)	17 (45.9)	20 (54.1)		
>400 thousand	28 (5.0)	10 (35.7)	18 (64.3)		
Inconvenient to disclose	51 (9.2)	14 (27.5)	37 (72.5)		

### Information, motivation, and behavioral skills

#### Construct validity

Estimated parameters for the measurement models (standardized estimated factor loadings, standard errors, and *R*^2^ values) are presented in Table 2. All items were significantly loaded onto the corresponding latent factors, with *R*^2^ ranging from 0.055 to 0.908. The construct validity of the IMB model components was assessed through the confirmatory factor analysis. The model fit indices indicated an acceptable model fit: (1) Information: χ^2^/df = 2.276 < 3, CFI = 0.998 > 0.90, and RMSEA = 0.048 < 0.08; (2) Motivation: χ^2^/df = 2.589 < 3, CFI = 0.998 > 0.90, and RMSEA = 0.053 < 0.08; and (3) Behavioral skills: χ^2^/df = 2.911 < 3, CFI = 0.998 > 0.90, and RMSEA = 0.059 < 0.08. Therefore, the measured items were acceptable.

**Table 2:** Standardized estimated factor loadings, standard errors, and *R*^2^ values of the IMB model constructs.

Variables	Estimated	S.E.	*p* value	*R* ^2^
1. Information				
Item 1	0.812	0.028	<0.001	0.660
Item 2	0.832	0.027	<0.001	0.693
Item 3	0.870	0.022	<0.001	0.756
Item 4	0.869	0.023	<0.001	0.755
Item 5	0.934	0.013	<0.001	0.872
Item 6	0.953	0.012	<0.001	0.908
Item 7	0.940	0.013	<0.001	0.884
Item 8	0.948	0.013	<0.001	0.899
Item 9	0.910	0.019	<0.001	0.828
Item 10	0.891	0.020	<0.001	0.795
Item 11	0.916	0.018	<0.001	0.839
2. Motivation				
Item 12	0.749	0.091	<0.001	0.562
Item 13	0.826	0.100	<0.001	0.682
Item 14	0.941	0.112	<0.001	0.885
Item 15	0.268	0.049	<0.001	0.072
Item 16	0.235	0.059	<0.001	0.055
3. Behavioral skills				
Item 17	0.846	0.018	<0.001	0.715
Item 18	0.846	0.019	<0.001	0.716
Item 19	0.861	0.018	<0.001	0.741
Item 20	0.863	0.018	<0.001	0.745
Item 21	0.714	0.024	<0.001	0.510

#### Correlations among components in the IMB model

The median (25–75th quartile) scores were 8.0 (3.0–11.0) for information, 17.0 (16.0–20.0) for motivation, and 20.0 (16.0–24.0) for behavioral skills. The univariate analyses found that the participants who ever did cancer screening significantly had better information or behavioral skills for cancer screening participation (*p* < 0.01; Table 3). The bivariate correlations among the IMB model constructs showed that all the variables were significantly associated with the others (Table 4).

**Table 3:** Univariate analyses of the associations between IMB model constructs and cancer screening behavior.

Variables	Ever did cancer screening			
	Yes [Median (25–75th quartile)]	No [Median (25–75th quartile)]	*Z*	*p* value
Information score	11.0 (7.0–11.0)	6.0 (1.0–11.0)	8.21	<0.001
Motivation score	17.0 (17.0–21.0)	17.0 (16.0–20.0)	1.27	0.220
Behavioral skills score	20.0 (19.0–25.0)	18.0 (15.0–22.0)	6.56	<0.001

**Table 4:** Spearman’s correlation analysis of the IMB model constructs.

Variables	Information	Motivation	Behavioral skills
1. Information	1		
2. Motivation	0.223^**^	1	
3. Behavioral skills	0.380^**^	0.205^**^	1
4. Ever did cancer screening	0.349^**^	0.113^**^	0.278^**^

**p < 0.01.

### The IMB model estimation

**Figure 1:**
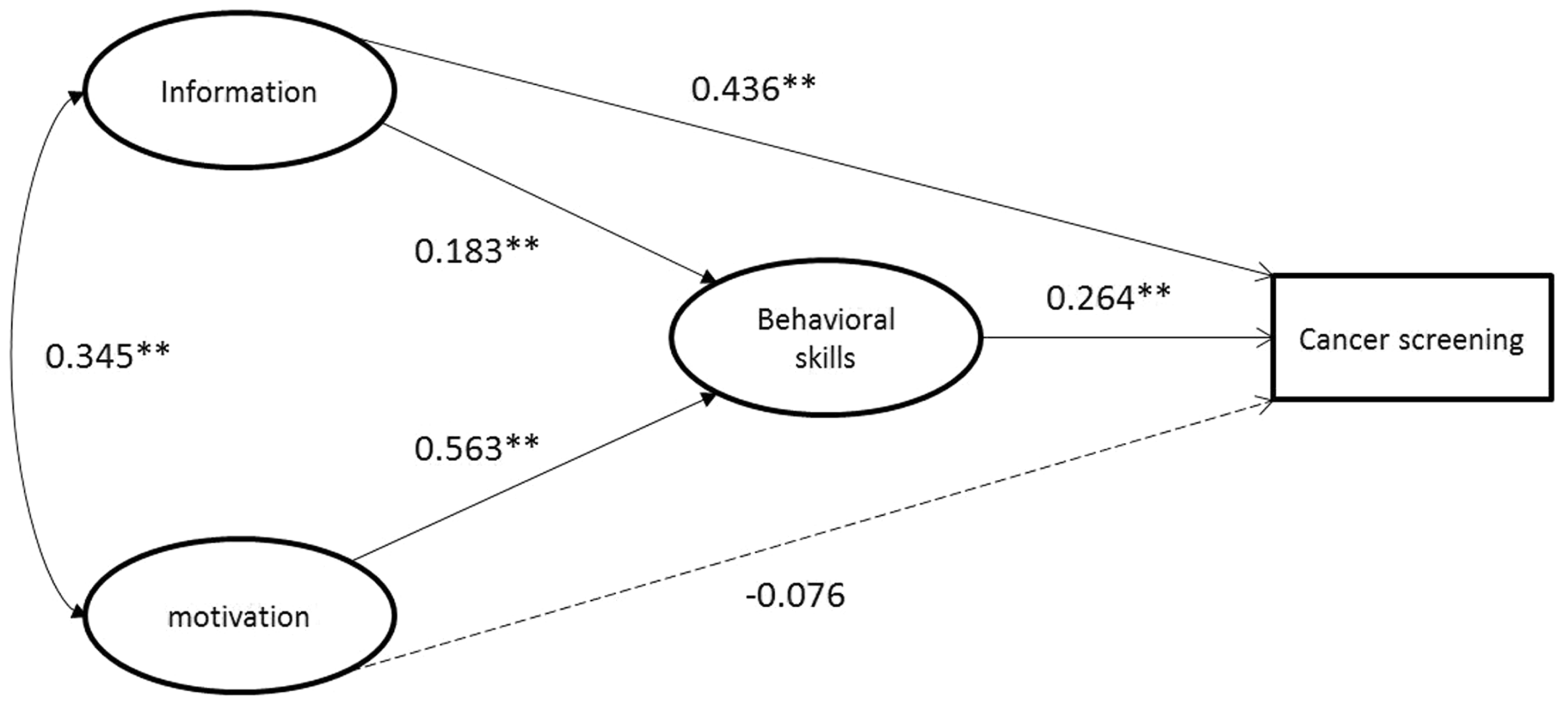
Estimation of the information-motivation-behavioral skills model of cancer screening behaviors. ^*^p < 0.05; ^**^p < 0.01.

The model is shown in Figure 1. The model fit indices indicated an acceptable model fit: χ^2^/df = 437.42/192 = 2.28 < 3, CFI = 0.985 > 0.90, and RMSEA = 0.048 < 0.08. Whether to participate in cancer screening was positively associated with behavioral skills (β = 0.264, *p* = 0.003). Motivation had no direct effect on cancer screening participation but had a strong effect on behavioral skills (β = 0.563, *p* < 0.001), which in turn significantly influenced the cancer screening behavior. The mediation test found that the indirect effects of motivation mediated cancer screening behavior through behavioral skills were 0.149 (95% CI = 0.057–0.288). Additionally, knowledge of cancer and its screening was positively associated with behavioral skills (β = − 0.075, *p* = 0.372) and cancer screening behavior (β = 0.052, *p* = 0.540). Information and motivation were significantly co-varied (*r* = 0.345, *p* < 0.001). In combination, these variables accounted for 30.4% of the variation in participating cancer screening. Path coefficients were showed in detail (Table 4).

## Discussion

In this study, the cancer screening rate of the working population in Shanghai was 34.4%. The three most common reasons for not participating in cancer screening were: (1) I am healthy and do not need screening; (2) I am too busy; and (3) I do not know the professional channels for screening. This rate was much lower than the cancer screening rates in European countries (72.4%) (26) and the United States (39.0, 22.2, 57.1, and 36.3% for cervical, colorectal, breast, and prostate cancers, respectively, between 2019 and 2021) (27). It was also much lower than that in the Korean population (28) and in East Asian communities. However, this discrepancy may primarily stem from the relatively younger age distribution observed in our study sample. The rate from this study was slightly higher than the rate from other urban communities in China (29, 30), which may have been due to variations in cancer screening items in the medical examination program for certain occupations.

Not surprisingly, those who underwent cancer screening possessed better knowledge of cancer warning signs and symptoms. Knowledge of workers who did not previously undergo cancer screening was only about 55% correct in this study. Awareness of symptoms increases the likelihood of participation in cancer screening by being vigilant to cancer when these conditions appear in oneself. Additionally, previous studies found that knowledge of cancer screening increased participation rates in a variety of screening behaviors, including breast self-exam and mammography (31), cervical smear test (32), and colorectal fecal occult blood test or colonoscopy (33, 34).

Inconsistent with our hypothesis, motivation was not directly associated with cancer screening behavior. The effect of motivation for participating in cancer screening was fully mediated by behavioral skills. Motivation is only one of the three components in the IMB model, and it may interact with information and behavioral skills in complex ways. Individuals who are highly motivated to engage in cancer screening may still need access to accurate information and sufficient behavioral skills to actually perform the screening behavior. A systematic review found that indigenous peoples with a poor attitude toward cancer screening were more likely to refuse cancer screening (35). Another study found that motivation factors increased willingness for an HPV test in United States women (36). Workplace support and family encouragement were also effective strategies for increasing the rate of screening participation (37, 38). Another explanation is that a part of the working population has multiple avenues for engaging in cancer screening possibly. Even in the absence of strong motivation, they can still participate in cancer screening through annual company-sponsored physical examination.

Our results emphasized the critical role of behavioral skills in promoting cancer screening. These skills incorporated how to determine the risk of developing the disease, how to obtain screening information, making screening appointments, and handling discomfort and fear during screening. Although people know in advance the importance of cancer screening and have positive attitudes toward it, they are often unable to fulfill the behavior if they do not have the appropriate competencies to undertake it. Behavioral skills were improved when subjects had more information and better motivation related to cancer screening. Among them, efforts to improve positive attitudes toward cancer screening were more effective than providing information and knowledge. This outcome was consistent with the findings of Misovich et al. (39) and Kim et al. (21).

The IMB model was innovatively used in this study to elucidate the pathways influencing screening behaviors and the effect of determinants, to understand the current status of cancer prevention screening and barriers to screening in the working population in Shanghai, and to provide an evidence base for specific future intervention strategies for cancer screening. Previous studies showed that model-based interventions was more effective in enhancing screening behaviors compared to non-model-based interventions (40). Healthcare providers could tailor their interventions to the needs of each individual by identifying the specific information, motivation, and behavioral skills deficits. For example, if an individual lacks the necessary behavioral skills to undergo cancer screening, healthcare providers could provide practical guidance and support to help the patient overcome these barriers, which could involve information about where to get screened, how to prepare for the screening, and how to interpret the results. Policymakers could use the findings of the study to design workplace-based interventions that provide information, motivation, and behavioral skills support for cancer screening through various channels, such as workplace posters, educational sessions, and mobile health apps.

Increasing the screening rate of cancer prevention in the working population has certain advantages. First, people in this age range are mostly affected by a variety of cancers and screening aids in early diagnosis and treatment. The incidence of most cancers increases rapidly from the age of 40 and the majority of the working population is between the ages of 30 and 60. Therefore, the timing of participating in cancer screening is appropriate and the benefits are significant. Second, the workplace is a good organizational base to manage and implement the health promotion program. Implementing a health promotion program is less costly in workplaces, and more likely to be successful and have greater benefits than a program without workplace support (38). Therefore, increasing the rate of cancer screening among the working population will not only protect the health of employees and improve employees’ satisfaction and productivity but also reduce excessive medical expenses and financial losses due to illness and absences.

Several studies have used the HBM or TPB to examine predictors of cancer screening behavior in China (41–45). Compared to these studies, the current study adds to the literature by using the IMB model to examine predictors of cancer screening behavior among the working population in China. One key difference between the IMB model and other models is that the IMB model places greater emphasis on the importance of behavioral skills in predicting health behaviors such as cancer screening. By including a broader range of factors, the IMB model provides a novel perspective and a more comprehensive understanding of the complex interplay of cognitive and behavioral factors that influence health behavior.

There were limitations to this study that should be acknowledged. Firstly, we performed a cross-sectional survey and thus, we could not establish any causal relationship. It would be beneficial to conduct longitudinal studies to investigate the causal relationships between the IMB predictors and cancer screening behavior. Secondly, self-reported data about participating in cancer screening may have contained recall bias. Also, this study recruited subjects from a convenience sample, and a future study should be undertaken with stratified random sampling based on factors such as occupational category. Furthermore, this study was conducted with a self-designed questionnaire, which may have lacked some components. Last but not least, there is no information on sex and cancer specific screening participation. Future studies will further enhance the questionnaire or find a standardized scale, and focus on cancer specific screening behavior.

## Conclusion

The cancer screening rate was lower than expected in working population. The IMB model could be applicable to behavioral interventions for cancer screening in the Chinese working population. In addition to the knowledge of cancer warning signs and symptoms, it is important to enhance interventions on screening behavioral skills to improve the cancer screening participation rate of the occupational group. To improve behavioral skills, it may be more effective to increase motivation rather than simply providing information and knowledge.

## Data availability statement

The raw data supporting the conclusions of this article will be made available by the authors, without undue reservation.

## Ethics statement

The studies involving human participants were reviewed and approved by the Fudan University Shanghai Cancer Center Institutional Review Board. The patients/participants provided their written informed consent to participate in this study.

## Author contributions

ZW performed the statistical analysis and drafted the manuscript. ZW, CZ, JS, MM, YH, and YZ participated in the design of the study and revision of the paper. LZ, CZ, and ZW participated in investigation and the data collection. All authors contributed to the article and approved the submitted version.

## Funding

This work was supported by the Shanghai Engineering Research Center of Artificial Intelligence Technology for Tumor Diseases (19DZ2251800); the Shanghai Research Project on Aging and Maternal and Child Health (2020YJZX0206); and the Cancer Foundation of China (NH2018001).

## Conflict of interest

The authors declare that the research was conducted in the absence of any commercial or financial relationships that could be construed as a potential conflict of interest.

## Publisher’s note

All claims expressed in this article are solely those of the authors and do not necessarily represent those of their affiliated organizations, or those of the publisher, the editors and the reviewers. Any product that may be evaluated in this article, or claim that may be made by its manufacturer, is not guaranteed or endorsed by the publisher.
