# Supplementary material for: Predictors of cancer screening behavior of the working population in China based on the information-motivation-behavioral skills model
Source: Front Public Health. 2023 Jul 27;11:1112172. doi: 10.3389/fpubh.2023.1112172 (PMC10412821; doi:10.3389/fpubh.2023.1112172)
Supplement: Supplementary file 1 [file Data_Sheet_1.docx]

**Supplementary Table 1. Results of cancer warning signs and symptoms knowledge**

| Items | Correctness rate (%) |
| --- | --- |
| 1. Do you think an unexplained lump or swelling could be a sign of cancer? | 57.0 |
| 2. Do you think a rapid change in the appearance of a mole could be a sign of cancer? | 64.7 |
| 3. Do you think persistent unexplained pain could be a sign of cancer? | 55.2 |
| 4. Do you think a sore that does not heal could be a sign of cancer? | 62.8 |
| 5. Do you think persistent difficulty swallowing could be a sign of cancer? | 60.8 |
| 6. Do you think a persistent change in bowel or bladder habits could be a sign of cancer? | 67.8 |
| 7. Do you think a persistent cough, hoarseness, or blood in sputum could be a sign of cancer? | 64.4 |
| 8. Do you think abnormal hearing, nosebleeds, or headache could be a sign of cancer? | 64.2 |
| 9. Do you think unexplained vaginal bleeding, especially contact bleeding, could be a sign of cancer? | 61.9 |
| 10. Do you think painless hematuria or dyspareunia could be a sign of cancer? | 64.9 |
| 11. Do you think unexplained fever, malaise, or weight loss could be a sign of cancer? | 67.4 |

**Supplementary Table 2.** **Results of cancer screening motivation**

| Items | Mean | SD |
| --- | --- | --- |
| 1. I think cancer screening is important for cancer prevention. | 4.38 | 0.91 |
| 2. I think cancer screening is effective for cancer prevention. | 4.36 | 0.85 |
| 3. My family, friend, or doctor recommended cancer screening. | 4.31 | 0.92 |
| 4. I am worried about the bad results of cancer screening, so I do not want to do early screening. | 2.67 | 1.54 |
| 5. Going to the hospital for cancer screening is hassle and inconvenience. | 2.51 | 1.42 |

SD, standard deviation.

**Supplementary Table 3. Results of cancer screening behavioral skills**

| Items | Mean | SD |
| --- | --- | --- |
| 1. I have access to cancer prevention and early screening information (e.g., professional media and lectures). | 3.78 | 1.13 |
| 2. I can put correct knowledge about cancer prevention and early screening into action. | 3.91 | 1.05 |
| 3. I can determine that certain behaviors may cause cancer and recognize my risk of developing cancer. | 3.81 | 1.08 |
| 4. I can tolerate and overcome the discomfort and fear that may be associated with medical examinations. | 3.70 | 1.12 |
| 5. I can find a proper and professional medical examination facility for cancer screening when needed. | 4.02 | 1.01 |

SD, standard deviation.

**Supplementary Table 4&5. Recommended Cancer Screening Schedule for Women and Men**

**Recommended Cancer Screening Schedule for Women:**

| **Age Group** | **Recommendation** |
| --- | --- |
| 21-29 years | Cervical Cancer Screening: Once every 3 years after 3 consecutive years of Pap smear without abnormalities.  Breast Cancer Screening: Clinical breast examination every 1-3 years. |
| 30-39 years | Cervical Cancer Screening: Once every 3 years after 3 consecutive years of Pap smear without abnormalities.  Breast Cancer Screening: Clinical breast examination every 1-3 years.  Lung Cancer Screening: Baseline low-dose CT scan at age 30. |
| 40-49 years | Breast Cancer Screening: Clinical breast examination annually. Mammogram every 1-2 years.  Cervical Cancer Screening: Pap smear every 3 years |
| 45 years and above | Colorectal Cancer Screening: Fecal occult blood test (FOBT) annually. Colonoscopy every 10 years |
| 50 years and above | Cervical Cancer Screening: Pap smear every 3 years until age 65.  Breast Cancer Screening: Clinical breast examination annually and mammogram every 1 to 2 years  Colorectal Cancer Screening: Fecal occult blood test (FOBT) annually. Colonoscopy every 10 years  Stomach Cancer Screening: Gastroscopy every 5-10 years starting at age 50. |
| 75 years and above | Continued screening if in good health with a life expectancy of 10 years or more. Screening after age 85 is not recommended. |

**Recommended Cancer Screening Schedule for Men:**

| **Age Group** | **Recommendation** |
| --- | --- |
| 30-39 years | Lung Cancer Screening: Baseline low-dose CT scan at age 30. |
| 40-49 years | Prostate Cancer Screening: Serum PSA (Prostate-Specific Antigen) test every 2 years. |
| 45 years and above | Colorectal Cancer Screening: Fecal occult blood test (FOBT) annually. Colonoscopy every 10 years |
| 50-74 years | Prostate Cancer Screening: Serum PSA (Prostate-Specific Antigen) test every 2 years.  Colorectal Cancer Screening: Fecal occult blood test (FOBT) annually. Colonoscopy every 10 years  Stomach Cancer Screening: Gastroscopy every 5-10 years starting at age 50. |
| 75 years and above | Continued screening if in good health with a life expectancy of 10 years or more. Screening after age 85 is not recommended. |


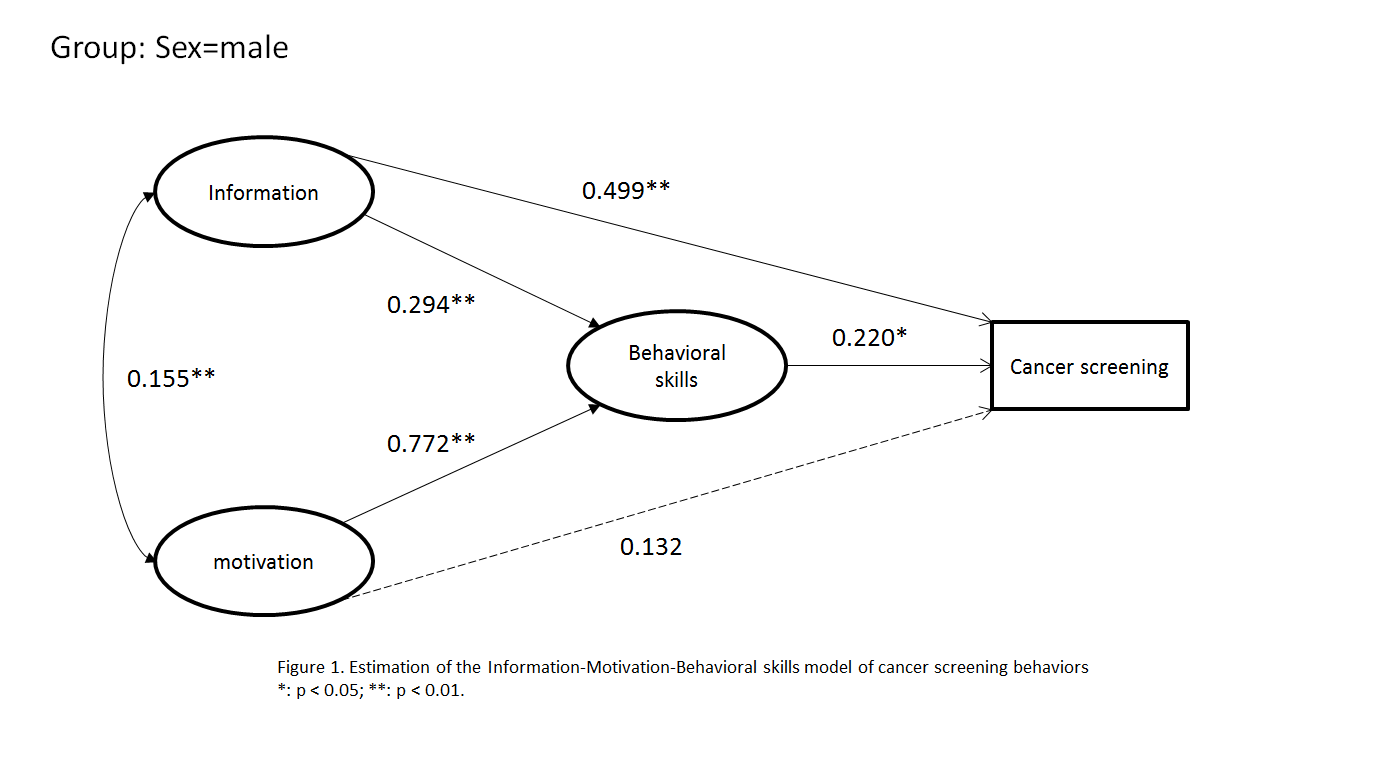


**Supplementary Figure 1. Estimation of the Information-Motivation-Behavioral skills model of cancer screening behaviors (Subgroup: Sex=male)**


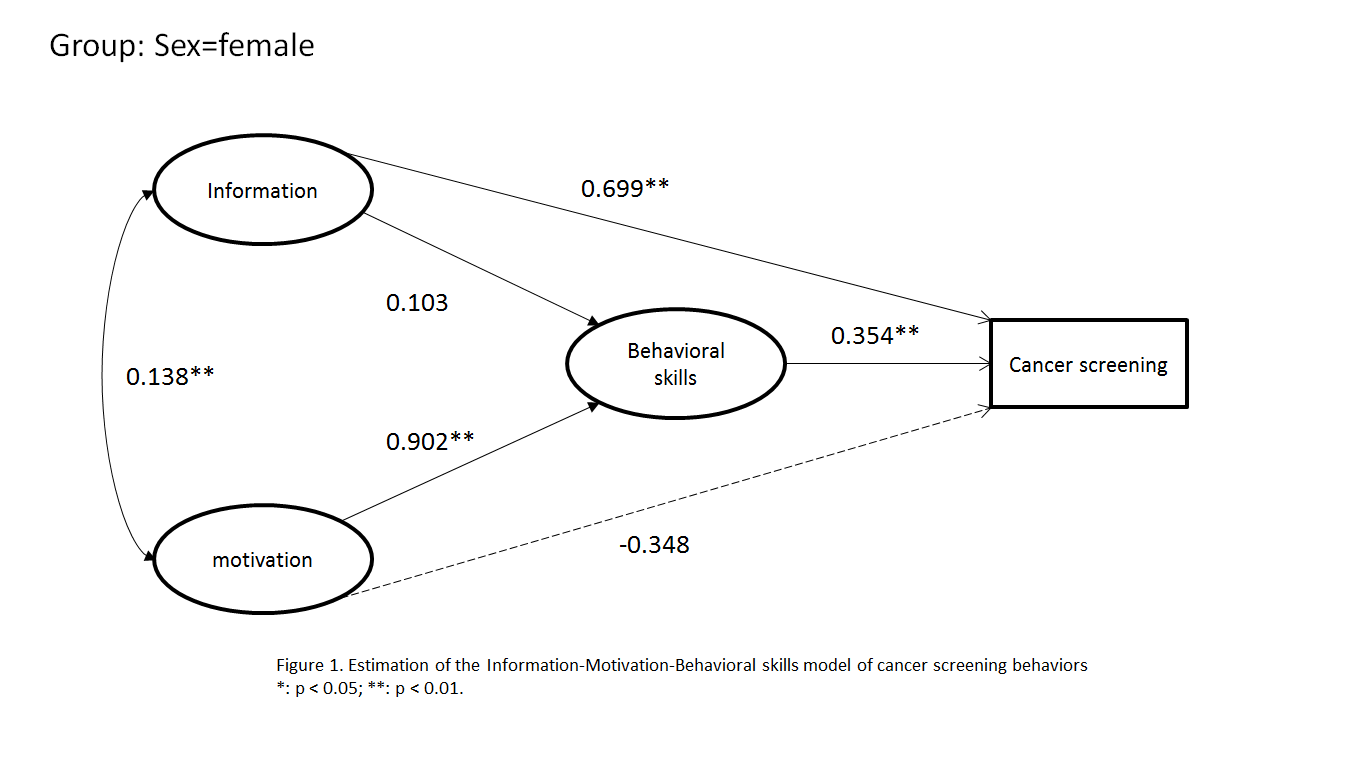


**Supplementary Figure 2. Estimation of the Information-Motivation-Behavioral skills model of cancer screening behaviors (Subgroup: Sex=female)**


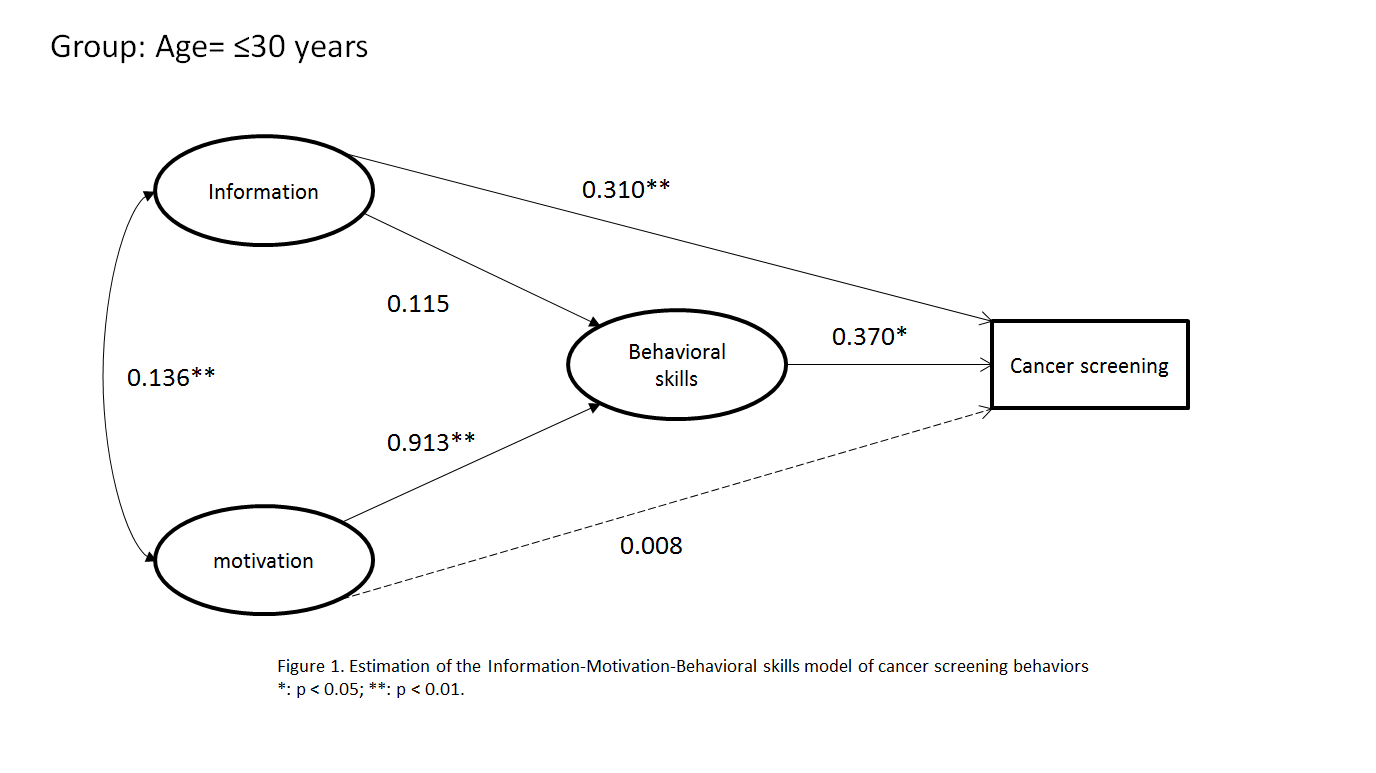


**Supplementary Figure 3. Estimation of the Information-Motivation-Behavioral skills model of cancer screening behaviors (Subgroup: Age= ≤30 years)**


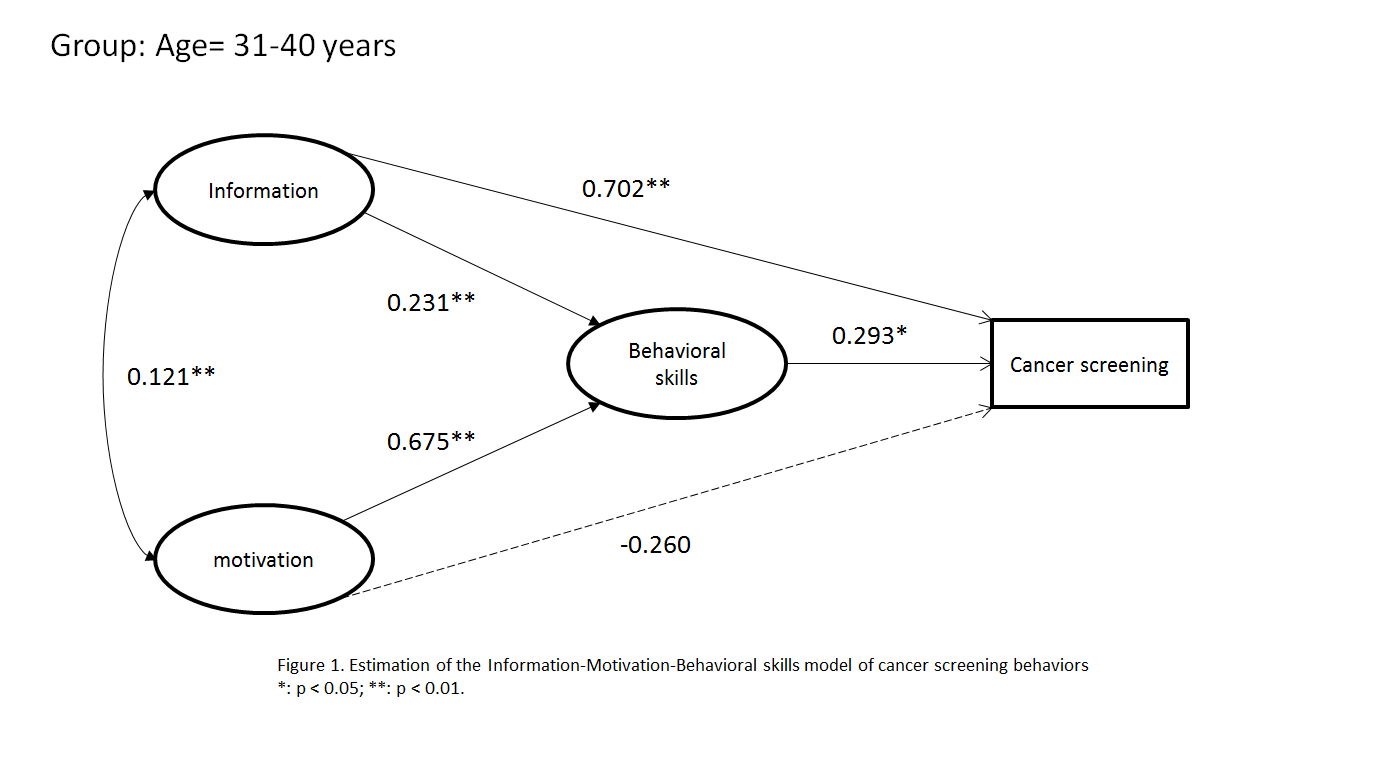


**Supplementary Figure 4. Estimation of the Information-Motivation-Behavioral skills model of cancer screening behaviors (Subgroup: Age= 31-40 years)**


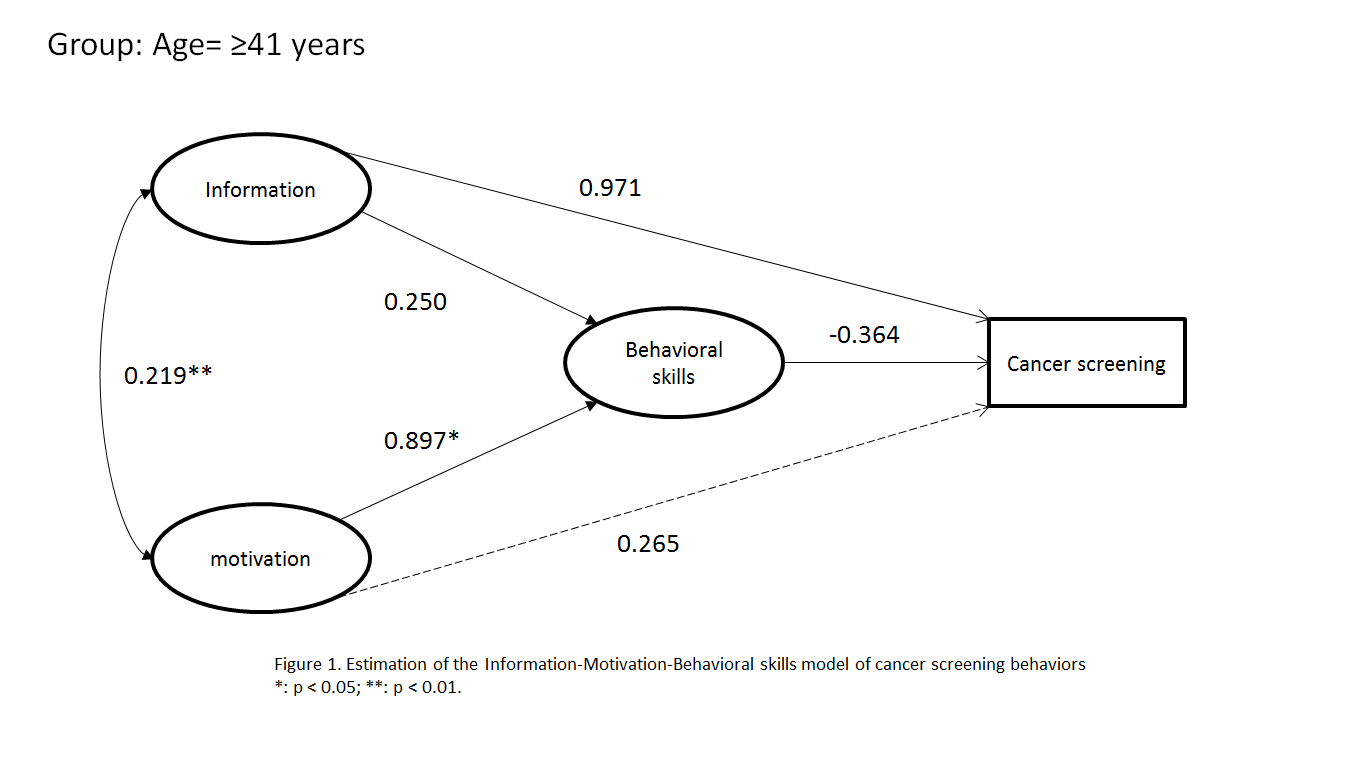


**Supplementary Figure 5. Estimation of the Information-Motivation-Behavioral skills model of cancer screening behaviors (Subgroup: Age= ≥41 years)**


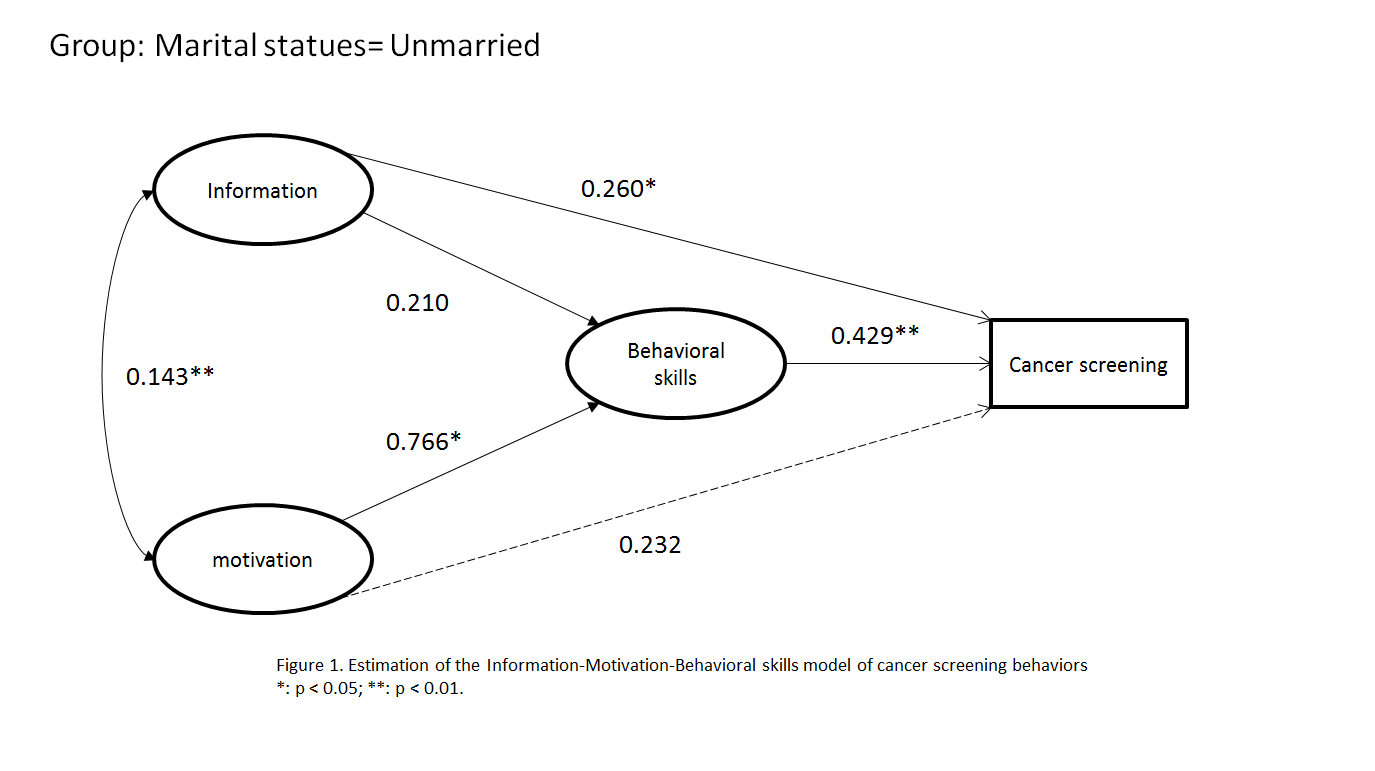


**Supplementary Figure 6. Estimation of the Information-Motivation-Behavioral skills model of cancer screening behaviors (Subgroup: Marital statues= Unmarried)**


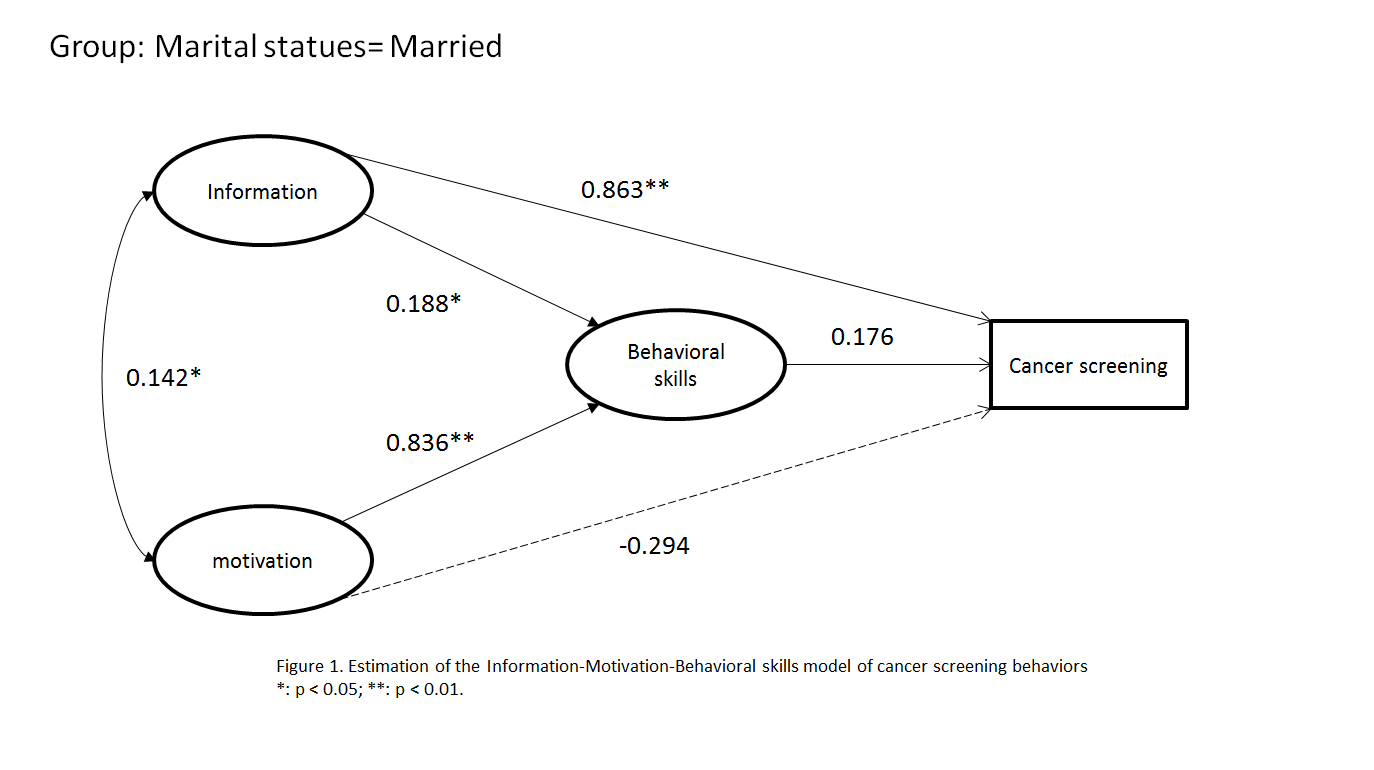


**Supplementary Figure 7. Estimation of the Information-Motivation-Behavioral skills model of cancer screening behaviors (Subgroup: Marital statues= Married)**


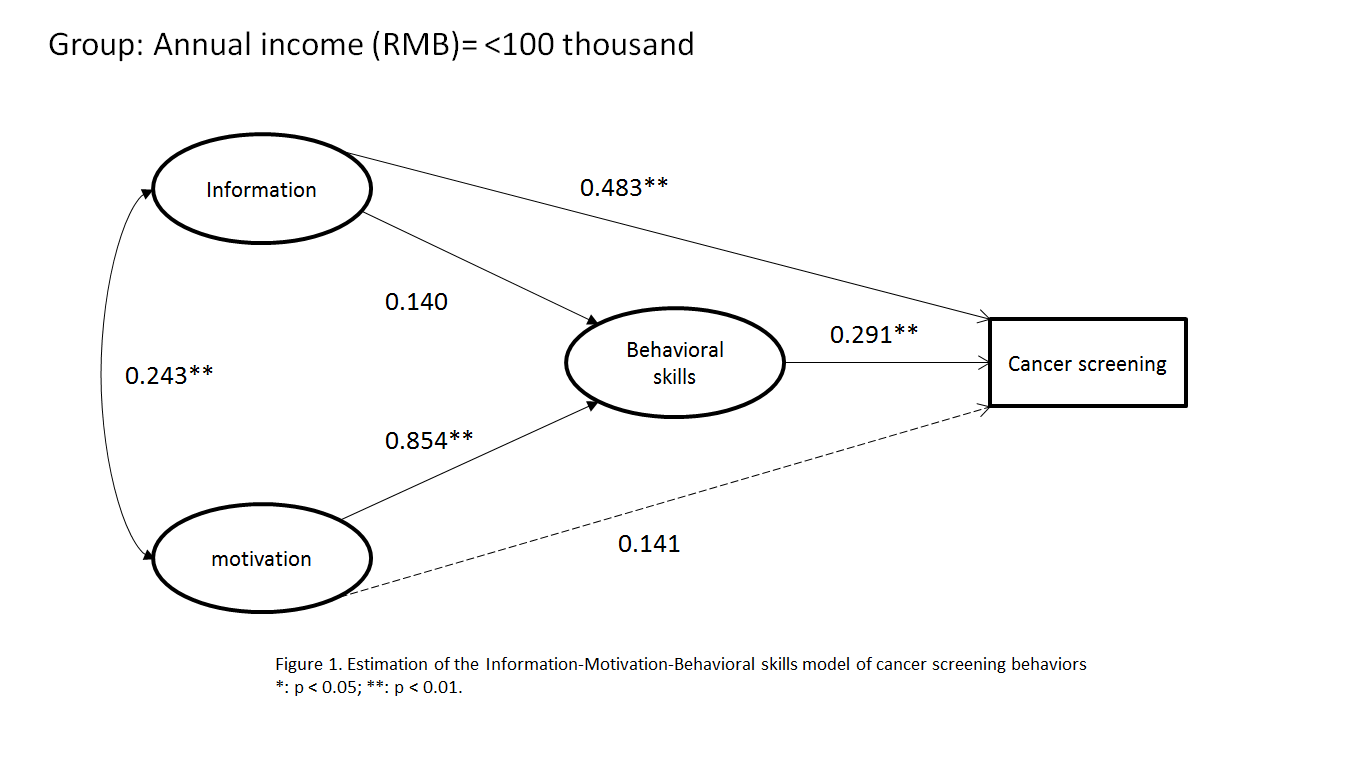


**Supplementary Figure 8. Estimation of the Information-Motivation-Behavioral skills model of cancer screening behaviors (Group: Annual income (RMB)= <100 thousand)**


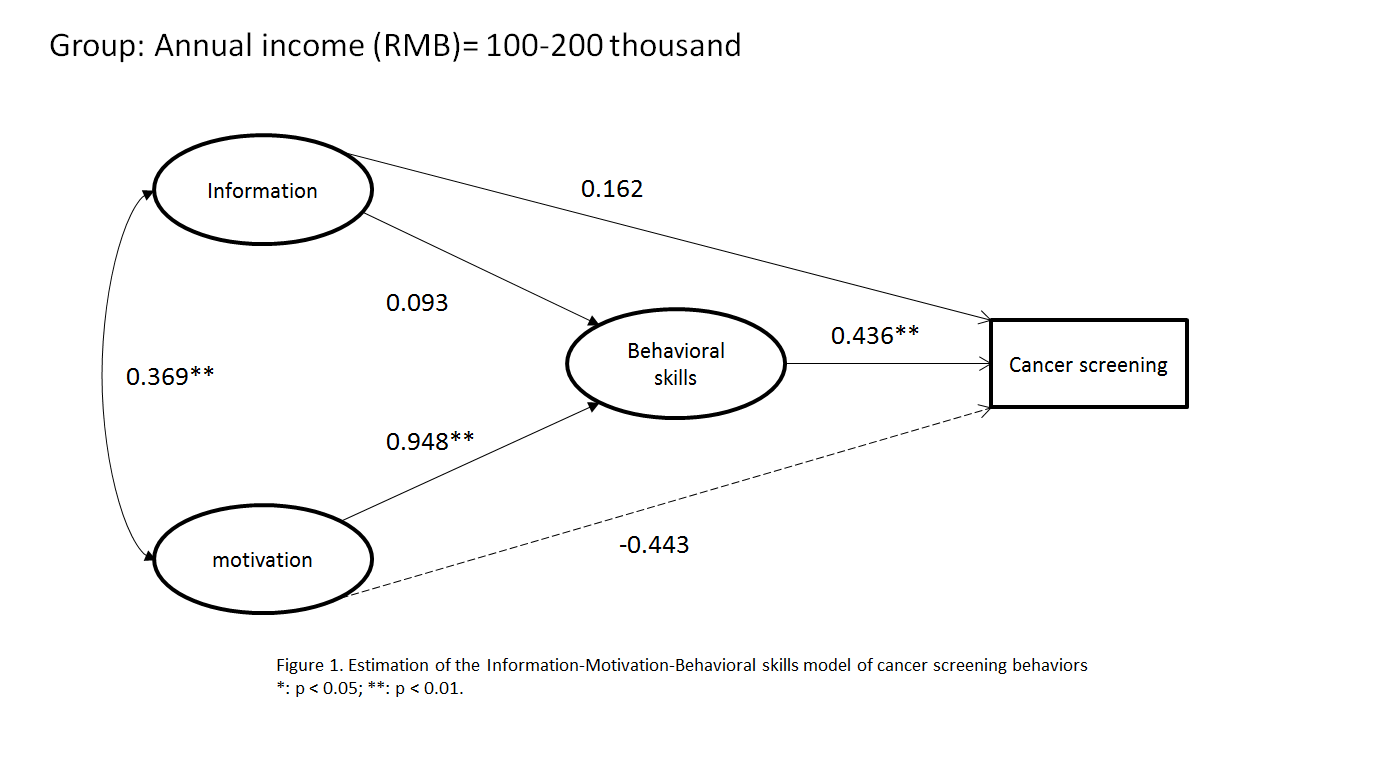


**Supplementary Figure 9. Estimation of the Information-Motivation-Behavioral skills model of cancer screening behaviors (Group: Annual income (RMB)= 100-200 thousand)**


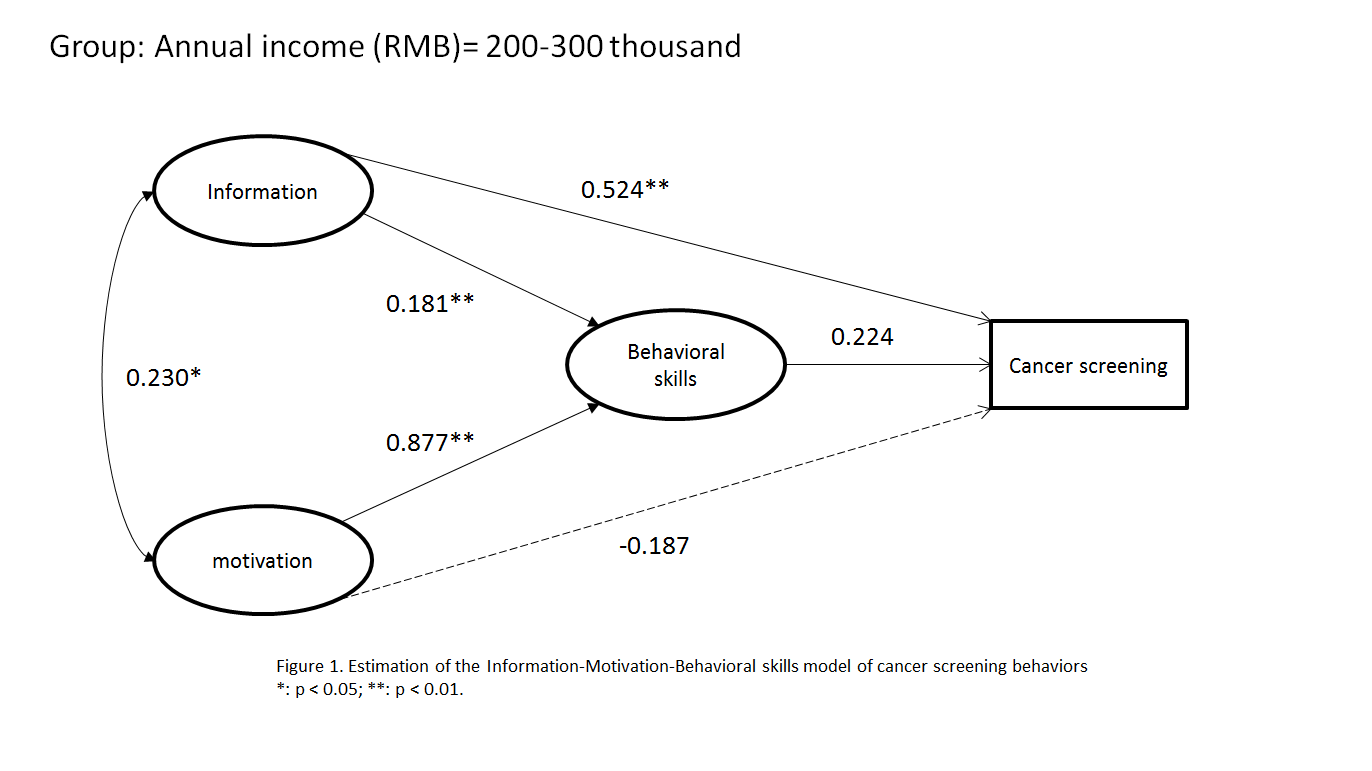


**Supplementary Figure 10. Estimation of the Information-Motivation-Behavioral skills model of cancer screening behaviors (Group: Annual income (RMB)= 200-300 thousand)**
